# Supplementary material for: Self-induced consensus of Reddit users to characterise the GameStop short squeeze
Source: Sci Rep. 2022 Aug 12;12:13780. doi: 10.1038/s41598-022-17925-2 (PMC9374300; doi:10.1038/s41598-022-17925-2)
Supplement: Supplementary file 1 — Supplementary Information. [file 41598_2022_17925_MOESM1_ESM.pdf]

Supplementary Information

Self-induced consensus of Reddit users to characterise the GameStop short squeeze

Anna Mancini[1,2,+], Antonio Desiderio[1,2,+], Riccardo Di Clemente[3,4,*] & Giulio Cimini[1,2,‡]

[1]*Physics Department and INFN, Unversità di Roma Tor Vergata, 00133 Rome (Italy)*

[2]*Centro Ricerche Enrico Fermi, 00184 Rome (Italy)*

[3]*Department of Computer Science, University of Exeter, (United Kingdom)*

[4]*The Alan Turing Institute, London NW12DB (United Kingdom)*

[*]*r.di-clemente@exeter.ac.uk*

[‡]*giulio.cimini@roma2.infn.it*

[+]*these authors contributed equally to this work*

Column	Description
Author	Username
Author ID	ID that uniquely identifies each Reddit user
Comment ID	ID that uniquely identifies each comment
Submission ID	ID of the post under which the comment was made
Parent ID	ID of the post or ID of the comment to which the given comment is a reply
Text	Text of the comment
UTC	Epoch Unix timestamp of the comment

Supplementary Table 1: Metadata downloaded from Pushshift for each Reddit comment.

Bot name
WSBVoteBot
RemindMeBot
Generic_Reddit_Bot
ReverseCaptioningBot
LimbRetrieval-Bot
NoGoogleAMPBot
RepostSleuthBot
GetVideoBot
CouldWouldShouldBot

Supplementary Table 2: Reddit bots removed from the database.

Column	Description
Time	Day timestamp of Stock Index
Open	Daily Opening Value of Stock Index
Close	Daily Closing Value of Stock Index
High	Daily High Value of Stock Index
Low	Daily Low Value of Stock Index
Volume	Daily Volume of Stock Transactions

Supplementary Table 3: Metadata downloaded from `polygon.io` for each stock ticker.

Supplementary Note 1: Lifetime and ramification of trees

To study how long a WSB conversation thread may last, we consider for each tree how many comments are written on each day from the creation of the root post onwards. As shown in the left panel of Supplementary Figure 1, most trees have a lifetime of one day, due to Reddit giving more relevance and visibility to newly written posts rather than older ones. Yet in some cases a small trail of comments can last even many days after the creation of the post. Another interesting tree feature to look at is the percentage of leaf comments (*i.e.*, comments without a further reply), which is inversely related to the height of the tree (*i.e.*, the maximal length of a branch). As shown in the right panel of Supplementary Figure 1, the number of leaves increases substantially in January, pointing to a less-structured conversation in this month. This pattern can be explained by users commenting mostly to cheer each other on during the short squeeze, contributing to a collective discussion rather than establishing structured one-to-one conversations [1, 2].

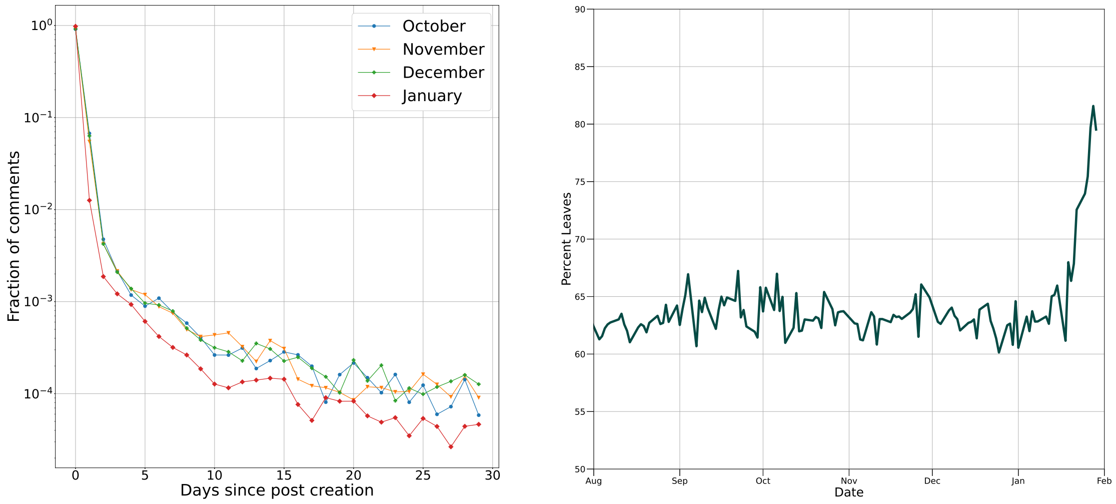

Supplementary Figure 1: (Left panel) Fraction of comments written on each day from the creation of the root post onwards, averaged over all trees generated in a given month. (Right panel) Time evolution of the fraction of leaf comments, averaged over all trees of a given day.

Supplementary Note 2: Ticker occurrences

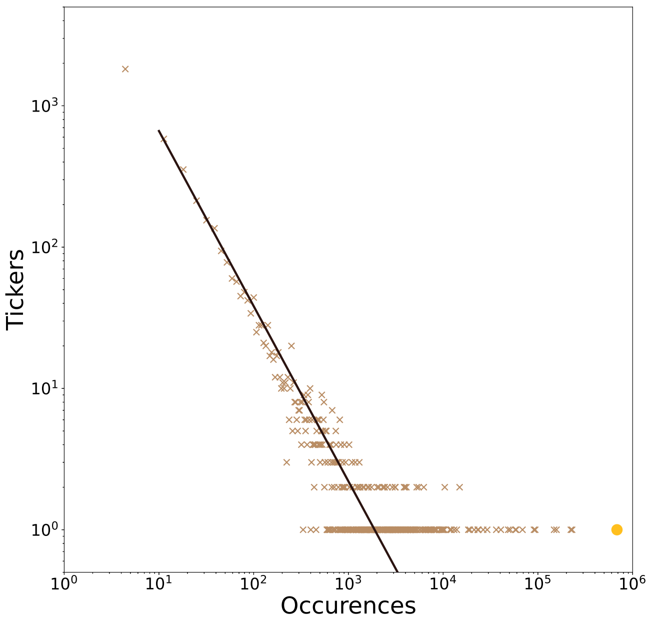

Supplementary Figure 2: Histogram of the total number of occurrences of the various stock tickers in the text of WSB posts and comments, from October 2020 to January 2021. 'GME' (in yellow) is the most frequent ticker. The black line has slope -1.23.

Supplementary Note 3: Modified VADER lexicon

As pointed out by other studies before ours, "VADER tends to overpredict neutral sentiment for WSB conversations, especially for posts labeled as 'positive' by human annotators. We speculate that this is due to a high proportion of out-of-vocabulary words [...] which VADER generally scores as neutral, as well as in-vocabulary words with WSB-specific senses that convey different sentiment polarity or intensity than they would in a generic social media context." [3]. A possible solution is to manually assign weights to several idiosyncratic slang-origin terms that are popular on WSB [4]. We followed this route by adding to the VADER lexicon a new group of words reported in Supplementary Table 4 below, together with their associated scores.

Word	Score	Emoji
rocket	4.0	yes
moon(ing)	4.0	no
diamond	4.0	no
gem stone	4.0	yes
hold(ing)	4.0	no
tendies	4.0	no
yolo	4.0	no
retard(s-ed)	2.0	no
autist(s)	2.0	no
degenerate(s)	2.0	no
ape(s)	2.0	no
gorilla(s)	2.0	yes
bear(s)	-2.0	no
paper	-4.0	no

Supplementary Table 4: Words (with possible suffixes) and corresponding emojis added to the VADER lexicon.

To demonstrate the importance of adapting VADER to the WSB jargon, we show in Supplementary Figure 3 the mean sentiment of comments containing 'GME', computed both using the modified lexicon and the original dictionary. The growing trend of sentiment is observed only in the former case (the same reported in Figure 2C of the main text), confirming that the words added to the VADER lexicon have a key role in revealing this signal. Note that the use of such jargon by WSB users is an intrinsic feature of the community and does not represent an innovation related to the GME event. To support this statement we report, on the same figure, the following quantities (computed daily): the frequency of comments containing at least one jargon term (*i.e.*, any of the words of the modified lexicon) and the overall frequency of such terms over all words used within WSB conversations. These frequencies remain fairly constant over time, with two exceptions: the modest peak at the end of November and the large increase at the end of January – which is however mainly due to the term 'hold(ing)' and occurs much after the growth of the mean sentiment. This evidence suggests that it is not the modified lex-

icon alone that causes the increase of sentiment, but the fact that these words are increasingly associated with text of positive polarity and related to ‘GME’. More importantly, this analysis ensures that the observed sentiment pattern is not biased by possible changes or innovations in the jargon of WSB users during the time window considered.

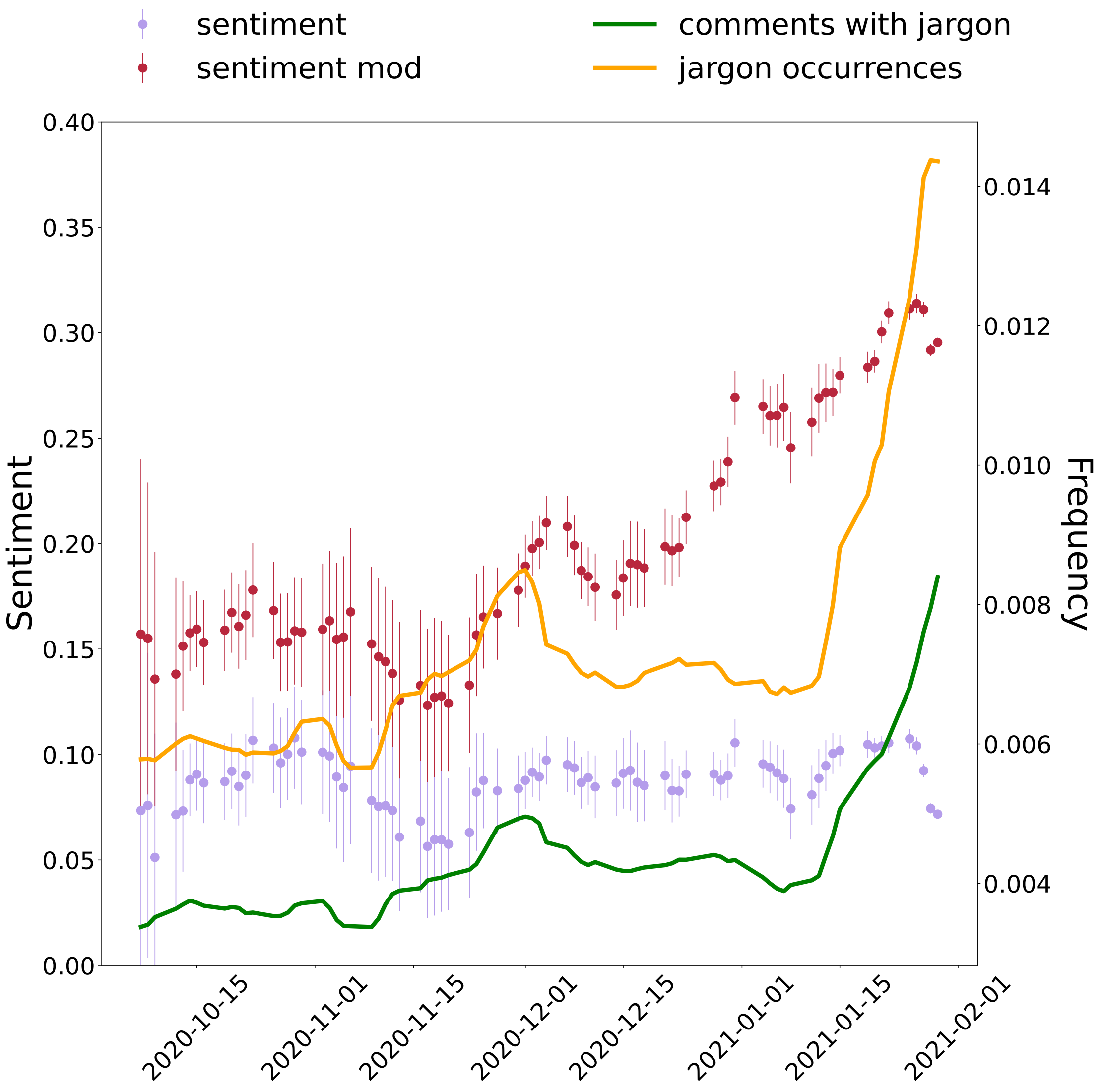

Supplementary Figure 3: Mean sentiment (and standard deviation of the mean) of comments containing ‘GME’, computed with the original VADER dictionary (violet points) or using the modified lexicon (red points, same of Figure 2C in the main text); Frequency of comments containing any of the words of the modified lexicon of Supplementary Table 4 (solid green line) and overall frequency of jargon words within all WSB conversations (solid orange line).

Supplementary Note 4: Hysteresis as a finite time phenomenon

Here we study the model dynamics at finite time horizon, *i.e.*, before the system may reach its equilibrium state. This is done by simulating the model for T time steps at a given value of c; the final state of this simulation is then used as the starting configuration for another simulation with a different value of c, and so forth. We change c in steps of 0.1, both in increasing (*forward*) and decreasing (*backward*) order. Supplementary Figure 4 reveals the presence of a hysteresis region in the phase transition, which becomes broader for small λ (*i.e.*, stronger network effects), meaning that in this region the system keeps a long memory of its previous state. This phenomenon is relevant for systems that are inherently out-of-equilibrium, like the process of opinion formation on a social network. In fact, the hysteresis disappears if T is large enough to allow the system to reach its equilibrium state $|m^*|$.

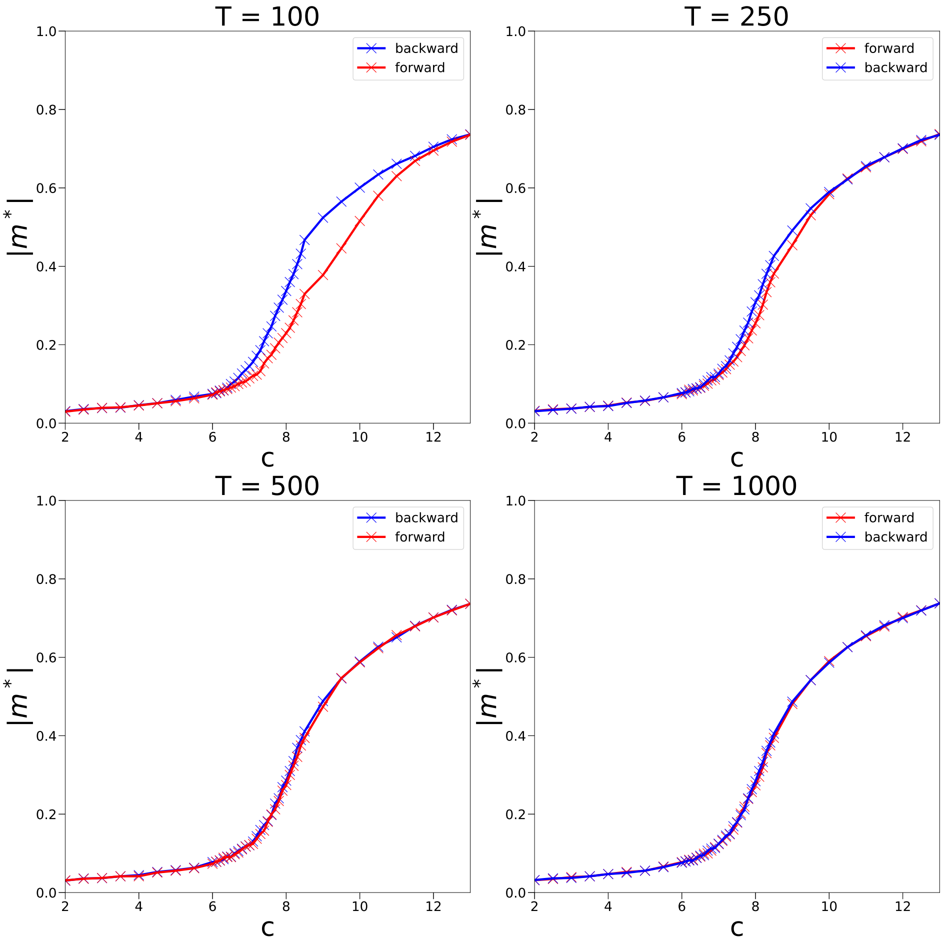

Supplementary Figure 4: Magnetization $|m^*(T)|$ averaged over 1000 runs of model simulations on Erdös-Rényi networks with $N = 10000$ nodes and average degree $\langle k \rangle = 20$, with a finite time horizon T.

Supplementary Note 5: Model simulations for varying λ

The value of λ sets how important is the local peer interaction with respect to the global action of the self-induced field. As shown by Figure 3D in the main text, in the case of Erdös-Rényi networks, for high values of $\lambda > 0.1$ we observe that the mean-field solution properly describes the critical behavior of the system regardless of the topological details of the underlying network. Instead for low values $\lambda < 0.1$ there are consistent deviations due to the structure of the network. The same observations hold for model simulations on empirical user-user networks: we report in Supplementary Figure 5 the cases $\lambda = 0.05$ (left panel) and $\lambda = 0.3$ (right panel), while the plot of Figure 4B in the main text refers to $\lambda = 0.1$.

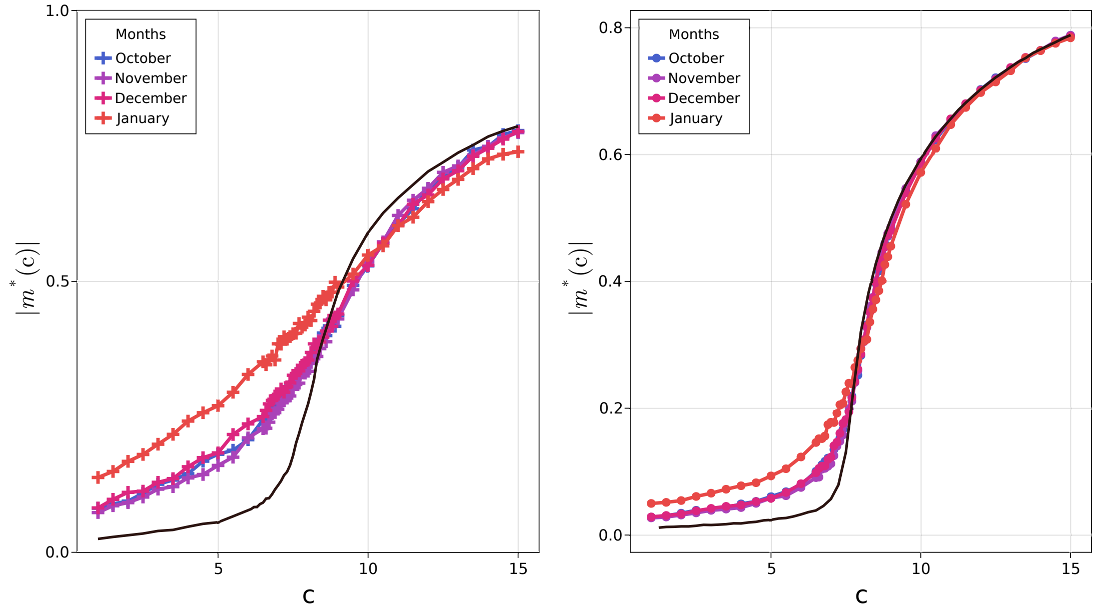

Supplementary Figure 5: Phase transition of the magnetization, for $\lambda = 0.05$ (left panel) and $\lambda = 0.3$ (right panel) obtained by simulating the model on the monthly networks, as compared to the transition observed on Erdös-Rényi graphs (black line).

Supplementary Note 6: Empirical evidence of the model

As discussed in the main text, empirical validation of the proposed Voter-like framework is not possible using solely WSB conversation data, for two reasons: i) we do not know how to map sentiment/engagement to the control parameter, and ii) since the user opinion (the order parameter) corresponds to participation or not to the short squeeze, we would need to know users' purchase transaction data, which are obviously not available. We can however make a more quantitative comparison between model and data following two directions.

Firstly we can provide evidence of the peer imitation dynamics at the basis of the Voter model, using the sentiment of conversations among interacting users (rather than their opinion, for which we have no data). In particular we select in our conversation data all 'reply-to' instances, where a 'child' comment directly replies to a 'parent' post or comment, such that either the parent or the child contains a 'GME' string. We then obtain the following counts: n_{++} is the number of parent-children pairs having both positive sentiment, n_{--} is the number of pairs with both negative sentiment, n_{+-} is the number of pairs where the parent has positive and the child negative sentiment, n_{-+} is the number of pairs where the parent has negative and the child positive sentiment, with $n_{TOT} = n_{++} + n_{--} + n_{+-} + n_{-+}$. We then compute the probability that the child comment is of the same sentiment polarity as the parent comment, namely $(n_{++} + n_{--})/n_{TOT}$, as well as the probability that the child comment is of the opposite polarity of the parent comment, given by $(n_{+-} + n_{-+})/n_{TOT}$. As reported in the left panel of Supplementary Figure 6, the fraction of concordant pairs is constantly much higher than that of discordant pairs, meaning that the copying mechanism is supported by data. This happens despite the fact that the mean sentiment grows in time (Figure 2C in the main text), as well as the probability of generating a positive child over that of generating a negative one, reported in the right panel of Supplementary Figure 6. Such an increasing tendency towards the positive polarity is what we argue is responsible for triggering the emergence of the self-induced field.

Secondly, we can assume to know a mapping between the mean sentiment and the control parameter c, and use such relation to compare the corresponding magnetization to the only empirical data available to proxy the real opinion of users: the price of GME shares. Of course the outcome of such comparison will strongly depend on the mapping assumed; however, as Supplementary Figure 7 shows, it is possible to obtain a good agreement by using a simple linear ansatz. We remark again that this exercise is solely intended to show that the model features an abrupt formation of consensus that is qualitatively similar to the GME price change due to the short squeeze.

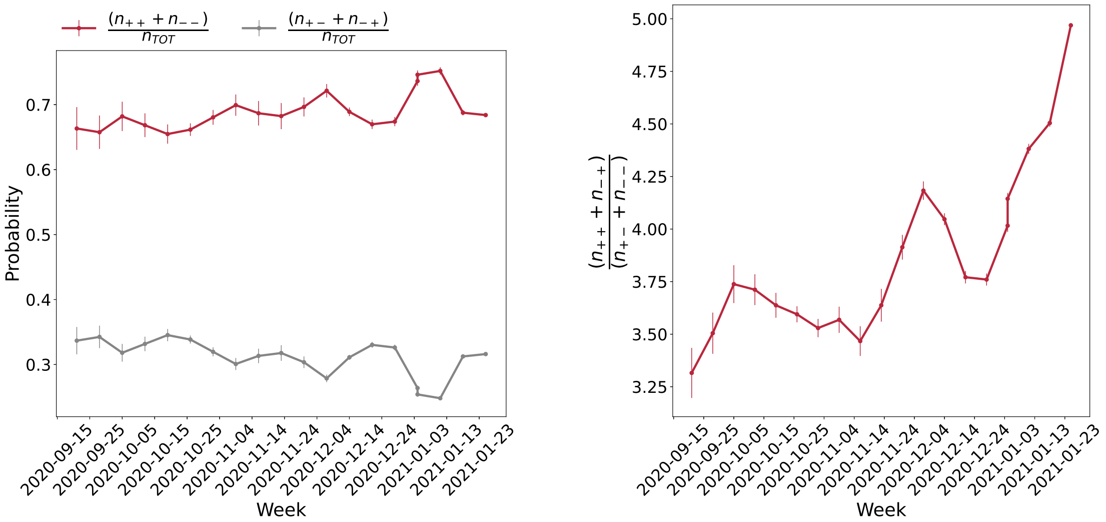

Supplementary Figure 6: (Left panel) Probability that the child comment is of the same or opposite sentiment polarity of the parent comment, respectively $(n_{++} + n_{--})/n_{TOT}$ and $(n_{+-} + n_{-+})/n$. (Right panel) Probability of generating a positive child over probability of generating a negative one, namely $(n_{++} + n_{-+})/(n_{+-} + n_{--})$. In both panels, error bars are obtained by assigning a Poissonian variance to the weekly counts.

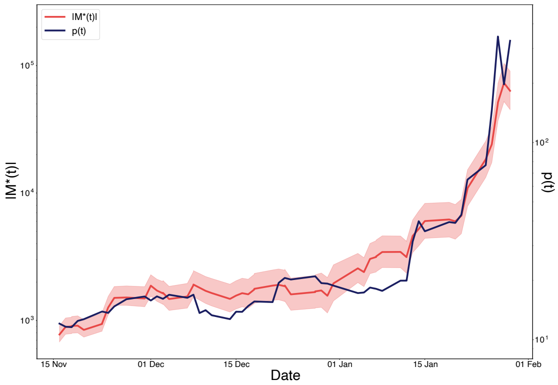

Supplementary Figure 7: Bayesian linear regression fit of the extensive magnetization, $|M(t)|$, versus the price of GME shares, $p(t)$, obtained according to the following steps. We select mean sentiment data on GME (those reported in Figure 2C of the main text) from mid November to end of January, and linearly fit the growing trend to obtain the linear interpolation of the sentiment, $\zeta(t)$. We then make the assumption $c(t) = a + b\zeta(t)$, and compute the corresponding $|m(t)|$ by interpolating the curves of the magnetization profiles in the various monthly networks, shown in Figure 4B of the main text. Here we show the plot of $p(t)$ versus $|M(t)| = |m(t)|N(t)$, where $N(t)$ is the number of active users (Figure 4C of the main text). Optimal fit parameters are obtained using a NUTS sampler with 10000 iterations, using a Gaussian priors for all variables.

Supplementary Note 7: Mean-field solution of the Voter model with self-induced field

We consider N users distributed over the nodes of a network. Each user i can assume two states, $s_i \in \{-1, +1\}$, that correspond to two different opinions (such as *join the short squeeze* or *do nothing*). The dynamics takes place as follows. Initially each opinion is set to $s_i = \pm 1$ with equal probability. At each time step t, a given individual i is selected at random and

- with probability $1 - \lambda$, she follows the usual voter dynamics and copies the opinion $s_j(t)$ of a randomly selected neighbor j (out of her k_i neighbors);
- with complementary probability λ, she follows a global field that is self-induced by the global state of the community (see below), and takes the opinion $e(t) = \pm 1$ assuming positive value with a probability $P_1[e(t)]$.

In formula:

$$s_i(t + \delta t) = \begin{cases} e(t) \text{ with probability } \lambda \\ s_j(t) \text{ with probability } \frac{1-\lambda}{k_i}, \end{cases} \tag{S1}$$

where $\delta t = 1/N$ and j is one of the neighbors of i. We assume that

$$P_1[e(t)] = \frac{c^{m(t)}}{1 + c^{m(t)}} \tag{S2}$$

where $m(t) = \frac{1}{N}\sum_i s_i(t)$ is the magnetization (*i.e.*, the average opinion) and $c \geq 1$ is the control parameter that sets how easily users tend to align with $m(t)$ (Supplementary Figure 8, upper left panel). Indeed for $c = 1$ we get $P_1[e(t)] = P_{-1}[e(t)] = 1/2$: the global field is pure noise and the model is equivalent to the noisy voter model. Instead when $c > 1$, $P_1[e(t)]$ is larger than $1/2$ for $m > 0$ and smaller for $m < 0$ (and quickly converges to ± 1 for $c \gg 1$, respectively).

The evolution of a system following eq. (S1) depends on the topology of the network defining the interactions among users, since the probability of being in a particular state depends on the state of neighboring nodes. We can however study the system in the *mean field approximation*, which assumes that neighboring states are independent. In this case the conditional probability of finding a neighbor in a particular state given the state of the selected node can be approximated by the fraction of users in that state out of the entire population. In practice this is equivalent to considering a complete graph structure [5].

We denote by $N_\uparrow$ the number of users in state $+1$, while $N_\downarrow = N - N_\uparrow$ is the number of users in the opposite state:

$$\begin{aligned} N_\uparrow(t) &= \sum_i \left(\frac{1 + s_i(t)}{2}\right) = \frac{N}{2}[1 + m(t)] \\ N_\downarrow(t) &= \sum_i \left(\frac{1 - s_i(t)}{2}\right) = \frac{N}{2}[1 - m(t)] \end{aligned} \tag{S3}$$

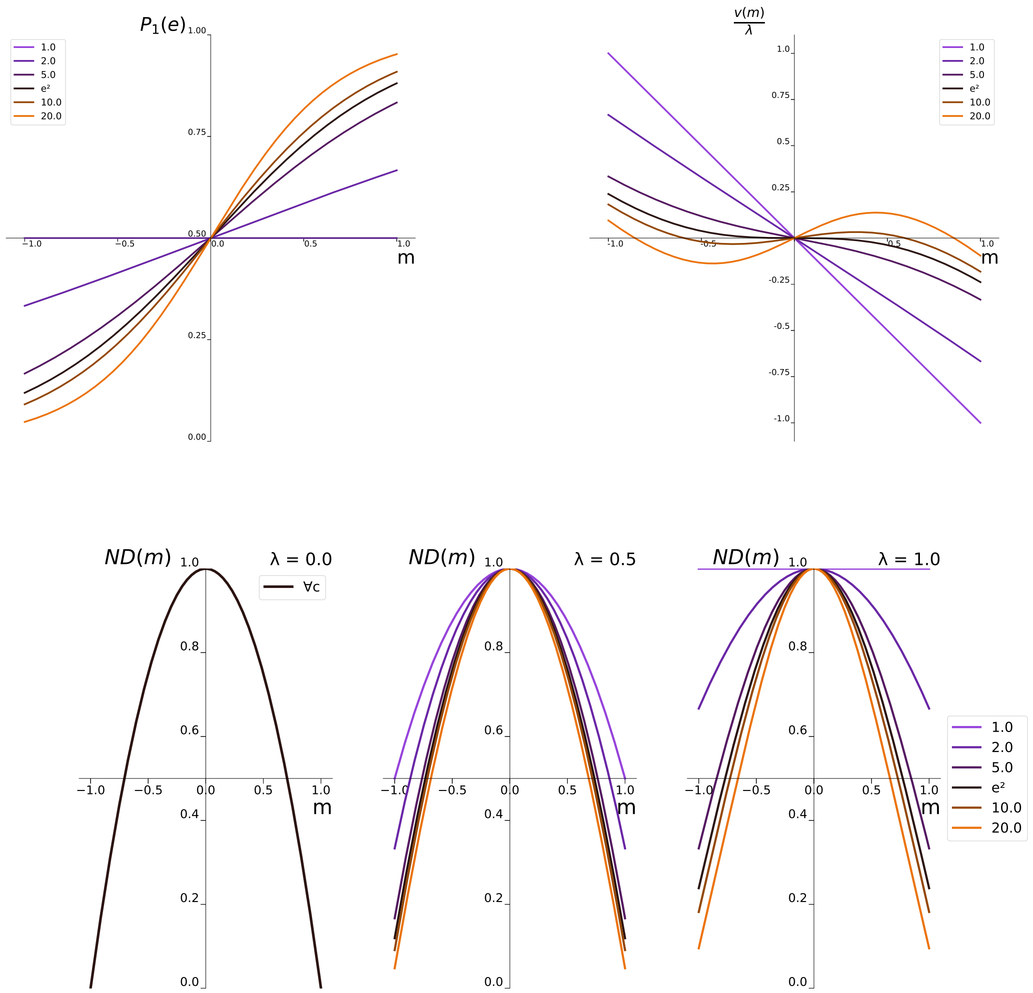

Supplementary Figure 8: (Upper panels) $P_1[e(t)]$ and drift coefficient $v(m)$ rescaled by λ, as a function of m for different values of c. (Lower panels) Diffusion coefficient $D(m)$ as a function of m for different values of c and λ.

Then the updating rule of eq. (S1) becomes

$$s_i(t+\delta t) = \begin{cases} e(t) & \text{with probability} \quad \lambda \\ +1 & \text{with probability} \quad (1-\lambda)\frac{N_\uparrow(t)}{N} \\ -1 & \text{with probability} \quad (1-\lambda)\frac{N_\downarrow(t)}{N} \end{cases} \tag{S4}$$

hence each time node i is selected her opinion evolves according to

$$s_i(t) \to s_i(t+\delta t) = \begin{cases} +1 & \text{with probability} \quad (1-\lambda)\left[\frac{1+m(t)}{2}\right] + \lambda P_1[e(t)] \\ -1 & \text{with probability} \quad (1-\lambda)\left[\frac{1-m(t)}{2}\right] + \lambda\{1 - P_1[e(t)]\} \end{cases} \tag{S5}$$

We now drop the explicit dependence of quantities on t. The probability of a spin-flip for a single user i is given by

$$\begin{aligned} R(s_i = -1 \to s_i = +1) &= \frac{1}{N}\left(\frac{1-s_i}{2}\right)\left[(1-\lambda)\frac{1+m}{2} + \lambda P_1(e)\right] \\ L(s_i = +1 \to s_i = -1) &= \frac{1}{N}\left(\frac{1+s_i}{2}\right)\left[(1-\lambda)\frac{1-m}{2} + \lambda\left(1 - P_1(e)\right)\right] \end{aligned} \tag{S6}$$

where the prefactor stems from the fact that the ith spin is selected with probability $1/N$. Summing the probabilities over all users we get the transition rates for the magnetization:

$$\begin{aligned} R(m) &= \left[(1-\lambda)\left(\frac{1-m}{2}\right)\left(\frac{1+m}{2}\right) + \lambda\left(\frac{1-m}{2}\right)P_1(e)\right] \\ L(m) &= \left[(1-\lambda)\left(\frac{1+m}{2}\right)\left(\frac{1-m}{2}\right) + \lambda\left(\frac{1+m}{2}\right)(1 - P_1(e))\right] \end{aligned} \tag{S7}$$

In the thermodynamic limit $N \to \infty$ the probability density $P(m,t)$ of a voter model dynamics evolves according to a diffusion process described by the Fokker-Plank equation [6], whose drift and diffusion coefficients are

$$\begin{aligned} v(m) &= \frac{\delta m}{\delta t}\left[R(m) - L(m)\right] \\ D(m) &= \frac{\delta m^2}{2\delta t}\left[R(m) + L(m)\right] \end{aligned} \tag{S8}$$

Considering that a single update occurs in a time $\delta t = \frac{1}{N}$ and the variation of m in a time step is equal to $\delta m = \frac{2}{N}$, we can substitute eqs. S7 into eqs. S8 and obtain

$$\begin{aligned} v(m) &= \lambda\left[f_c(m) - m\right] \\ D(m) &= \frac{1}{N}\left\{(1-\lambda)(1-m^2) + \lambda\left[1 - m f_c(m)\right]\right\} \end{aligned} \tag{S9}$$

where

$$f_c(m) = 2P_1(e) - 1 = \frac{c^m - 1}{c^m + 1} \tag{S10}$$

If $P(m,t)$ follows a Fokker-Plank equation then the corresponding value of m evolves according to a stochastic differential equation of the form [7]

$$dm = v(m)dt + \sqrt{D(m)}dW \tag{S11}$$

where dW is the standard Wiener process. Concerning the drift coefficient (Supplementary Figure 8, upper right panel) we have $v(m) = 0$ for $\lambda = 0$; otherwise it scales linearly with

λ. For $c = 1$ the drift has sign opposed to m, hence the system is always driven towards the stable point $m = 0$. However for growing c the drift decreases, and after the threshold value c^* the point $m = 0$ becomes unstable and a stable point $|m^*| > 0$ appears. Instead the diffusion coefficient (Supplementary Figure 8, bottom panels) is always $O(1/N)$ when $m \simeq 0$, and can be neglected otherwise unless c and λ are both close to 1. However the drift term plays the key role of driving the system out of the initial equilibrium state $m = 0$.

We can study the system around $m \simeq 0$ (*i.e.*, the initial configuration) using the first order approximation $f_c(m) \simeq m \ln \sqrt{c}$. In this case the stochastic differential equation for m becomes

$$\begin{aligned} dm(t) &\simeq -\lambda(1 - \ln\sqrt{c})m\,dt + \sqrt{\frac{1 - m^2[1 - \lambda(1 - \ln\sqrt{c})]}{N}}\,dW \\ &\simeq -\lambda(1 - \ln\sqrt{c})m\,dt + \left(1 - \frac{m^2}{2}[1 - \lambda(1 - \ln\sqrt{c})]\right)\frac{dW}{\sqrt{N}} \end{aligned} \tag{S12}$$

and we can see how the drift term changes sign for $c = e^2$. By discarding terms of order $O(m^2)$ we are left with

$$dm(t) \simeq -\lambda m(1 - \ln\sqrt{c})dt + \frac{dW}{\sqrt{N}} \tag{S13}$$

which represents an Ornstein-Uhlenbeck process whose formal solution, given $m(0) = 0$, is

$$m_t = \frac{e^{-\lambda(1-\ln\sqrt{c})t}}{\sqrt{2N\lambda(1 - \ln\sqrt{c})}} W_{e^{2\lambda(1-\ln\sqrt{c})t}-1} \tag{S14}$$

We can see now how the critical value $c = e^2$ corresponds to a Weiner process (the traditional purely diffusive voter dynamics), which characterizes the transition between the disordered and ordered phases.

References

[1] Toshio Yamagishi and Toko Kiyonari. The group as the container of generalized reciprocity. *Social Psychology Quarterly*, 63(2):116–132, 2021/11/22/ 2000.

[2] Lorenzo Lucchini, Luca Maria Aiello, Laura Alessandretti, Gianmarco De Francisci Morales, Michele Starnini, and Andrea Baronchelli. From Reddit to Wall Street: The role of committed minorities in financial collective action. *Royal Society Open Science*, 9(4):211488, 2022.

[3] Charlie Wang and Ben Luo. Predicting $GME stock price movement using sentiment from Reddit r/wallstreetbets. In *Proceedings of the Third Workshop on Financial Technology and Natural Language Processing*, pages 22–30, 2021.

[4] Abhinav Anand and Jalaj Pathak. The role of Reddit in the GameStop short squeeze. *Economics Letters*, 211:110249, 2022.

[5] Claudio Castellano, Santo Fortunato, and Vittorio Loreto. Statistical physics of social dynamics. *Reviews of Modern Physics*, 81(2):591–646, 05 2009.

[6] Pavel L. Krapivsky, Sidney Redner, and Eli Ben-Naim. *A Kinetic View of Statistical Physics*. Cambridge University Press, Cambridge, 2010.

[7] C. W. Gardiner. *Handbook of stochastic methods for physics, chemistry and the natural sciences*, volume 13 of *Springer Series in Synergetics*. Springer-Verlag, Berlin, third edition, 2004.
